# Evaluation and Transplantation of a SARS-CoV-2 Seropositive Kidney Candidate

**DOI:** 10.1155/2021/6613023

**Published:** 2021-03-08

**Authors:** Maya C. Graves, Sapna A. Mehta, Bonnie E. Lonze, Nicole M. Ali

**Affiliations:** ^1^New York University Grossman School of Medicine, New York, NY, USA; ^2^New York University Langone Transplant Institute, New York, NY, USA

## Abstract

The COVID-19 pandemic affected transplant center activity in areas with high number of cases such as New York City and prompted reevaluation of patients awaiting organ transplant diagnosed with SARS-CoV-2 infection. To resume safe transplantation at our center, we found it necessary to (1) identify transplant candidates with possible exposure to or history of COVID-19 infection, (2) outline a clinical and laboratory assessment to determine adequate clinical recovery from COVID-19 for transplantation, and (3) determine whether the possibility of perioperative COVID-19 transmission from the patient to staff would pose unacceptable risk. Here, we describe our center's approach to proceeding with transplantation in a SARS-CoV-2 seropositive living donor kidney transplant recipient and describe early posttransplant outcomes.

## 1. Introduction

**Figure 1:**
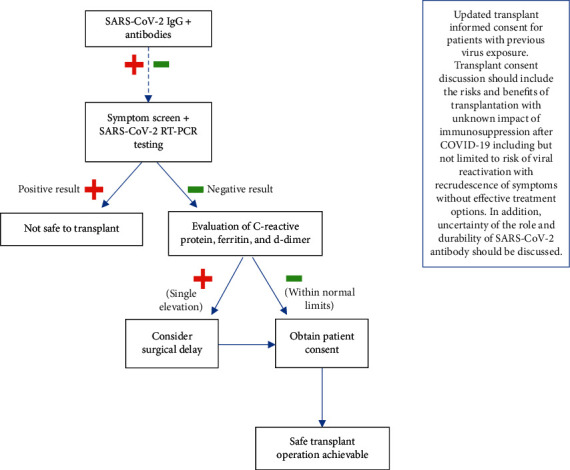
Protocol for transplant pre-operative evaluation for all transplant candidates during COVID-19 pandemic.

Kidney transplantation is a lifesaving treatment for many patients with end-stage renal disease (ESRD). The spread of the SARS-CoV-2 pandemic in areas such as New York City, where a surge in COVID-19 cases occurred, created a need to suspend solid organ transplantation when COVID-19-safe pathways for postoperative care were not available. While dialysis can be a temporary treatment option available to patients with kidney failure, this surrogate option is costlier and leads to higher morbidity and mortality rates than kidney transplantation [1]. Currently, there is limited guidance for safely transplanting patients during this COVID-19 era [2]. Additionally, kidney transplant candidates are at risk for exposure to COVID-19 while awaiting organ transplantation, particularly patients undergoing community-based hemodialysis. As the ability to safely resume transplantation from a hospital resource standpoint was regained, a new pretransplant framework was required to assess risk for patients exposed to or recovered from COVID-19. We describe a case of a living donor kidney transplant (LDKT) candidate found to be SARS-CoV-2 seropositive and the protocol we (Figure 1) utilized to assess the COVID-19 related risk to the recipient, donor, and provider care team which informed decision-making regarding timing of transplantation.

## 2. Case Report

A 30-year old female with a past medical history of hypertension, hyperlipidemia, polycystic ovarian syndrome, and focal segmental glomerulosclerosis presented with Stage IV chronic kidney disease (CKD) for an LDKT. Roughly, one year prior to presentation for the LDKT, she was evaluated by the transplant review committee to assess candidacy for a deceased donor kidney transplant (DDKT). She was approved and listed as active on the kidney transplant waiting list. During her waiting period, she never initiated dialysis. One year later, her kidney function was poor with creatinine elevated to 5.02 mg/dl, urine protein elevated to 265 mg/dL, BUN elevated to 43 mg/dl, and estimated GFR was 11 ml/min/1.73^2^. She was in imminent need for either transplantation or initiation of community-based hemodialysis. A living donor candidate was evaluated and approved to donate just prior to the outbreak of the COVID-19 pandemic. Months later, during routine preoperative evaluation, she underwent a complete COVID-19 symptom screen via telemedicine visit which was normal. During routine preoperative laboratory testing, the patient underwent SARS-CoV-2 IgG testing at an outside laboratory using the Abbott assay which resulted positive (23.88 UA/mL). Upon repeat discussion with the patient, she recalled that two months prior she had experienced 2 days of body aches and one day of fever with Tmax 38.6°C followed by two weeks of poor appetite. However, at the time of her symptoms, SARS-CoV-2 RT-PCR testing was not widely available and the patient had been tested only for influenza virus by nasopharyngeal swab which was negative. Her prolonged poor appetite was attributed to mild uremic symptoms from ESRD but improved without renal replacement therapy. The transplant team concluded that the patient had experienced a self-limited febrile illness during a period of known SARS-CoV-2 transmission in the New York City that was consistent with COVID-19 and that she had likely been infected.

Two days prior to scheduled transplant surgery, the patient underwent repeat symptom screen, reported no symptoms of COVID-19, and underwent SARS-CoV-2 RT-PCR testing by nasopharyngeal sample. In addition, as a further measure to assess potential patient-to-staff transmission risk, SARS-CoV-2 RT-PCR testing was performed on the patient's stool specimen and also resulted negative. The Cepheid Xpert Xpress SARS-CoV-2 assay was validated for both nasopharyngeal and stool samples by our institution's clinical laboratory. She was admitted for scheduled living donor kidney transplant surgery. She had normal vital signs, an unremarkable lung exam, and an admission chest X-ray showing clear lungs without signs of pneumonia. Repeat SARS-CoV-2 RT-PCR testing by nasopharyngeal swab was negative and laboratory values for inflammatory markers (ferritin, d-dimer, and C-reactive protein) were all within normal limits. The patient had an uncomplicated intra- and postoperative course. The kidney was flushed, per surgeon standard practice, with porcine heparin (2500 units in 500 mL normal saline), and the patient was given standard venous thromboembolism (VTE) prophylaxis. She was administered high-dose methylprednisone intraoperatively followed by basiliximab (20 mg) for induction therapy. Induction immunosuppression for transplant surgery was not modified based on the history of COVID-19. The patient was ambulating on postoperative day 1, serum creatinine continued to improve to normal, and urine output was excellent. She was discharged on postoperative day 4 on an immunosuppressive regimen of tacrolimus, mycophenolate mofetil, and prednisone. At the time of this report, she has completed 6 months of outpatient follow up with no signs or symptoms of infection, rejection, or other complications. She has undergone surveillance SARS-CoV-2 PCR testing at 0, 2, 4, 6, and 22 weeks after transplant, and the results have remained negative.

## 3. Discussion

This is the first reported case of kidney transplantation in a SARS-CoV-2 seropositive patient in the United States with 6 months of posttransplant follow-up. Transplant candidates with a history of clinical COVID-19 or seropositivity for SARS-CoV-2 are increasingly common as we move forward in the COVID-19 era [3–5]. As a transplant center located in the initial epicenter at the onset of the COVID-19 outbreak in the United States, we found it critical to create a systematic approach (Figure 1) to (1) identify whether transplant candidates had evidence of SARS-CoV-2 infection, (2) determine when the patient was adequately clinically recovered to proceed with transplantation and use of immunosuppression, and (3) assess risk of SARS-CoV-2 transmission from the patient to the clinical care team.

Three cardinal concerns were identified:

1. Confirmation of prior SARS-CoV-2 infection and assessment for active viral infection with transmission potential
2. Consideration of risk of recrudescence of COVID-19 symptoms or potential for SARS-CoV-2 reactivation with onset of immunosuppression, given the limited data to date
3. Adequate precautions for our surgeons, physicians, and all healthcare personnel caring for a seropositive patient in the peritransplant period

We addressed the first concern by thorough history including the review of any prior laboratory testing, COVID-19 symptom screening, use of validated SARS-CoV-2 serologic assay, and SARS-CoV-2 RT-PCR testing. Our patient was found to be seropositive for SARS-CoV-2 first at an outside laboratory which was utilized until a validated COVID-19 serologic assay was available at our hospital. Subsequently, when serologic testing became available at our transplant center, the test was performed on sera stored from the same date as the sample tested with the Abbott assay and was found to be positive, increasing our confidence in a true positive serology result. We assessed transmission potential by SARS-CoV-2 RT-PCR testing on nasopharyngeal and stool samples. Our rationale for utilizing both nasopharyngeal and stool RT-PCR testing was based on data in adults and children showing SARS-CoV-2 RT-PCR positivity in stool for a longer duration (>4 weeks) than nasopharyngeal or respiratory samples [6]. Given that 2 months had lapsed since our patient's COVID-19 compatible symptoms, our suspicion for positive nasopharyngeal RT-PCR testing was low. The RT-PCR Cepheid Xpert Xpress SARS-CoV-2 Assay was validated by our institution's clinical laboratory for both nasopharyngeal and stool samples. The utility of the RT-PCR SARS-CoV-2 assay under the Food and Drug Administration's Emergency Use Authorization in our institution's core-laboratory facilitated prompt turn-around times (<5 hours) amidst the pandemic, allowing for time sensitive decision-making regarding transplantation [7]. All transplant candidates at our center undergo SARS-CoV-2 PCR by nasopharyngeal swab testing prior to transplant surgery, regardless of the presence of COVID-19 symptoms or whether prior SARS-CoV-2 serology testing has been performed.

**Figure 2:**
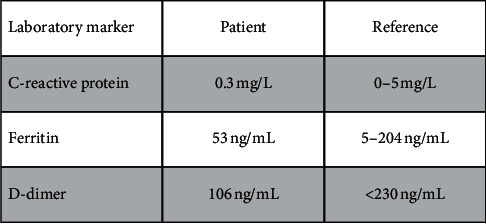
Inflammatory markers used to evaluate COVID-19 clinical recovery in SARS-CoV-2 seropositive transplant candidate.

The third element of laboratory data reviewed which provided confidence that our patient was clinically recovered from COVID-19 were inflammatory markers. C-reactive protein, d-dimer, and ferritin were reviewed as numerous studies, and meta-analyses have identified abnormalities in these values as dominant biomarkers for COVID-19 disease progression and poor outcome [8]. All values were within normal limits at the time of preoperative evaluation (Figure 2).

The long-term natural history of SARS-CoV-2 remains to be fully characterized, and the risk associated with induction immunosuppression soon after infection is unclear. While prolonged SARS-CoV-2 PCR positivity has been reported in patients following clinical recovery from COVID-19, persistence of replicative virus regardless of serial nasopharyngeal PCR results and risk of recrudescence of symptoms remains unclear. We felt it was prudent and ethically necessary to address this unknown and potential risk with the patient through a modification of informed consent for kidney transplantation. The risk versus benefit analysis when discussing the option to move forward with transplantation and initiation of life-long immunosuppression required patient acknowledgement of the unknown risks of a novel pathogen including, but not limited to, reactivation or recrudescence of COVID-19 symptoms, impact on the kidney allograft, and potential for poor long-term outcome. Additionally, the unknown durability and immunologic role of SARS-CoV-2 IgG were discussed.

During the kidney transplant procedure, safety precautions taken by the operating room team members included the use of an N95 or Delta mask for all operating room staff and face shields for those performing the orotracheal intubation. In accordance with algorithmic precautions for operating members during the COVID-19 pandemic set forward by Forrester et al., our risk assessment performed by symptomology, measurement of inflammatory markers, and utilization of repeat RT-PCR testing provided confidence that patient did not have evidence of ongoing nasopharyngeal viral shedding and that use of an N95 or Delta mask in combination with eye protection would be sufficient for healthcare worker protection [9].

Given the atypical nature of respiratory disease presentation in immunocompromised populations [10], close clinical surveillance for the recrudescence of COVID-19 symptoms in our patient remains important. Case series of kidney transplant recipients with COVID-19 have described variable initial clinical presentations, including gastrointestinal symptoms, vomiting, fever, cough, generalized fatigue, or chills [11–14]. To our knowledge, no cases of recrudescence of COVID-19 symptoms following organ transplantation have been described. After transplantation, our patient's clinical care has been managed via telemedicine every 2–4 weeks by transplant nephrology and with routine posttransplant weekly laboratory testing (complete blood count with differential, complete metabolic panel, magnesium, phosphorus, tacrolimus level, BK virus, and Epstein–Barr Virus, and a urinalysis). In addition, SARS-CoV-2 RT-PCR testing was repeated at weeks 0, 2, 4, 6, and most recently, 22 weeks after transplant. As recommended by the Centers for Disease Control and Prevention (CDC), the use of telemedicine has allowed for active monitoring of our patient while mitigating increased risk of repeat COVID-19 exposure for both patient and provider [15]. At 6 months of posttransplant follow-up, our patient has not encountered any complications suggestive of SARS-CoV-2 symptom recurrence, rejection, or other complications. We report favorable early outcomes after kidney transplantation in a recipient with recent SARS-CoV-2 infection and suggest development of a systematic risk assessment for organ transplantation after COVID-19. Further long-term data on posttransplant outcomes in COVID-19 recovered patients are needed.

## Data Availability

The data that support the findings of this study are available upon request from the corresponding author with permission from NYU Langone Health, New York. The data are not publicly available due to privacy and/or ethical restrictions.

## Abbreviations

CDC: Centers for Disease Control and Prevention
COVID-19: Coronavirus disease 2019
CKD: Chronic kidney disease
ESRD: End-stage renal disease
LDKT: Living donor kidney transplant
RT-PCR: Real-time reverse transcription polymerase chain reaction
SARS-CoV-2: Severe acute respiratory syndrome coronavirus 2.
